# Characteristics of drugs for ultra-rare diseases versus drugs for other rare diseases in HTA submissions made to the CADTH CDR

**DOI:** 10.1186/s13023-018-0762-1

**Published:** 2018-02-01

**Authors:** Trevor Richter, Ghayath Janoudi, William Amegatse, Sandra Nester-Parr

**Affiliations:** 1 CADTH, 865 Carling Ave., Suite 600, Ottawa, ON K1S 5S8 Canada; 2 Rare Access Ltd, 59 Grove Street, Leamington Spa, CV32 5AG England

**Keywords:** Rare diseases, Ultra-rare diseases, Orphan drugs, Technology assessment, health, Canada

## Abstract

**Background:**

It has been suggested that ultra-rare diseases should be recognized as distinct from more prevalent rare diseases, but how drugs developed to treat ultra-rare diseases (DURDs) might be distinguished from drugs for ‘other’ rare diseases (DORDs) is not clear. We compared the characteristics of DURDs to DORDs from a health technology assessment (HTA) perspective in submissions made to the CADTH Common Drug Review. We defined a DURD as a drug used to treat a disease with a prevalence ≤ 1 patient per 100,000 people, a DORD as a drug used to treat a disease with a prevalence > 1 and ≤ 50 patients per 100,000 people. We assessed differences in the level and quantity of evidence supporting each HTA submission, the molecular basis of treatment agents, annual treatment cost per patient, type of reimbursement recommendation made by CADTH, and reasons for negative recommendations.

**Results:**

We analyzed 14 DURD and 46 DORD submissions made between 2004 and 2016. Compared to DORDs, DURDs were more likely to be biologic drugs (OR = 6.06, 95%CI 1.25 to 38.58), to have been studied in uncontrolled clinical trials (OR = 23.11, 95%CI 2.23 to 1207.19), and to have a higher annual treatment cost per patient (median difference = CAN$243,787.75, 95%CI CAN$83,396 to CAN$329,050). Also, submissions for DURDs were associated with a less robust evidence base versus DORDs, as DURD submissions were less likely to include data from at least one double-blinded randomized controlled trial (OR = 0.13, 95%CI 0.02 to 0.70) and have smaller patient cohorts in clinical trials (median difference = −108, 95%CI –234 to −50). Furthermore, DURDs are less likely to receive a positive reimbursement recommendation (OR = 0.22, 95%CI 0.05 to 0.91), and low level of evidence was the major contributor for a negative recommendation.

**Conclusions:**

The results suggest that DURDs could be viewed as distinct category from an HTA perspective. Applying the same HTA decision-making framework to DURDs and DORDs might have contributed the higher rate of negative reimbursement recommendations made for DURDs. Recognition of DURDs as a distinct subgroup of DRDs by explicitly defining DURDs based on objective criteria may facilitate the implementation of HTA assessment process that accounts for the issues associated with DURD.

## Background

Treatments for rare diseases (RDs) are increasingly the focus of drug developers, as reflected by the strong market growth of the ‘orphan drug’ sector [1]. However, rare diseases technologies face key challenges in satisfying the expected evidence requirements of regulators, HTA agencies, and payers. Randomised controlled trials (RCTs), the gold standard for obtaining robust clinical evidence, are generally complex in RDs due to small patient numbers, and clinical evidence is typically limited to small, short-term trials, often relying on surrogate outcome measures [2]. The cost of drugs that treat RDs are usually high, as manufactures strive to recoup the drug development costs from a small target market [3]. In order to address the specific challenges posed by the limitations to generate robust evidence for RD treatments, regulators and HTA agencies have started to implement specific policies for the assessment of these technologies [1, 4]. However, no universal definition of ‘rare disease’ has emerged, and therefore no corresponding universal definition exists for ‘therapies for the treatment of rare diseases’ [5]. Current definitions of RD are based on arbitrary prevalence (or incidence) thresholds, and the most commonly used definitions require that a disease affect no more than 50 per 100,000 people to be considered a RD [5]. Definitions of RD based on such prevalence thresholds are often combined with additional descriptive components, such as diseases severity (5).

An RD prevalence threshold of, for example, ≤ 50 per 100,000 people equally includes diseases that affect as few as 1 per 100,000 individuals as well as much rarer diseases that affect only a few individuals per million. Therefore, populations with a disease defined as ‘rare’ by the aforementioned prevalence threshold may differ in size by 500%. In a previous paper, we described the characteristics of all drugs for RDs that fit the definition of a prevalence of less than 50 per 100,000 people [6]. Subsequent to the publishing of our paper, a letter to the editor reanalyzing our data contributed to the ongoing debate on whether extremely RDs should be recognized as being distinct from ‘other’, more prevalent, RDs and encouraged Canada to adopt a proper framework to deal with RDs [7]. Regulatory and HTA frameworks that recognise ultra-RD as a distinct category are already in operation in several European jurisdictions [8, 9]. Various definitions of ‘ultra-RD’ have been proposed and typically include prevalence thresholds that vary from 1 to 20 patients per million people (4). How treatments for ultra-RDs might be systematically distinguished from those that target more prevalent RDs has not been examined in depth.

In this study, we sought to empirically compare the key characteristics related to clinical data, drug cost, and rates of negative reimbursement recommendations of submissions for drugs to treat RDs (DRD) made to the CDR in Canada from 2004 until 2016. With this study, we aim to enrich the discussion on the potential need to recognise DURD as a separate category from other types of RD for HTA purposes.

## Main text

### Methods

We conducted a search to update the dataset of DRD that we collected and reported on in a previous paper [6]. Briefly, two reviewers (GJ and WA) independently screened all submissions to the CDR in Canada since 2004 (the start of the CDR process) and until 2016, inclusive. We extracted publicly available data from the CADTH website (www.cadth.ca) for all submissions made to the CDR in the study period. We excluded submissions for treatments of diseases with a prevalence of > 50 per 100,000 people. Where disease prevalence was not specified in a CADTH recommendation report, we sourced prevalence figures from Orphanet (www.orpha.net). Where a prevalence range was reported, we used the mean. Submissions that were withdrawn, under review at the time data collection, or for which a CDR recommendation was not available were also excluded.

For the purpose of this study, drugs for rare diseases (DRDs) were defined in line with the commonly used definition of DRDs, or ‘orphan drugs’, that treat diseases affecting ≤ 50 per 100,000 people [5]. We further subdivided DRDs into two mutually exclusive categories (see Table 1): drugs for ultra-RDs (DURDs) were defined as drugs used to treat ultra-rare diseases affecting ≤ 1 patient per 100,000 people, while all other DRDs, i.e. those for diseases affecting > 1 to 50 per 100,000 people, were classified as drugs for other rare diseases (DORDs). The threshold prevalence of ≤ 1 patients per 100,000 people that we used to categorize DURDs is similar to, but slightly narrower than that proposed in other definitions of ultra-RDs, such as the threshold of < 2 patients per 100,000 people applied to define a DURDs in England and Scotland [8, 9].

**Table 1:** Definitions of terminology used in the current study

Term	Definition
Drugs for rare diseases (DRDs)	Drugs used to treat diseases that affect ≤ 50 per 100,000 people.
Drugs for ultra-rare diseases (DURDs)	Drugs used to treat diseases that affect ≤ 1 per 100,000 people. -
Drugs for other rare diseases (DORDs)	Drugs used to treat rare diseases that affect > 1 to 50 per 100,000 people.

We extracted information relating to the following parameters from each submission that met the inclusion criteria:

#### Molecular basis of treatment

We determined whether the treatment was a small molecule or a biologic molecule, and what the mechanism of action is based on, e.g., whether the drug is an analog or an inhibitor of a particular molecule.

#### Prevalence

Prevalence was determined from the reccomendation report or, if not available, using values for the disease type obtained from Orphanet (orpha.net).

#### Characteristics of clinical data

The characteristics of clinical study data within each submission was determined based on several parameters, including the number of studies, study size, study types (double-blind vs. open-label), randomized vs. non-randomized, comparative vs. non-comparative, controlled vs. non-controlled), and whether an active comparator or placebo was included.

#### Cost

The average cost of treating one patient for 1 year was captured if this information was available publicly. Where price or annual treatment costs per patient were not available, we calculated an average annual treatment cost based on the unit cost cited in the submissions and dosing schedules specified in the relevant product monograph. Where unit cost was not available, a web search of Canada’s public plans formularies or the manufacturer media releases using the generic or the trade name of the drug was conducted to determine if a treatment cost in Canada was publicly available. In addition, published incremental cost per quality adjusted life year figures were collected from the CDEC recommendations.

#### Recommendation type

If the recommendation was to ‘list’, ‘list with criteria and/or conditions’, ‘reimburse’, or ‘reimburse with criteria and/ or conditions’, it was classified as positive. If the recommendations was to ‘do not list’, ‘do not list at the submitted price’, or ‘do not reimburse’ it was classified as negative.^1^ Reasons for a negative recommendation were assessed qualitatively and categorized into three distinct groups, specifically:

- Clinical only: in cases where high uncertainty and major limitations in the clinical evidence were the driving factors, or where there is methodologically sound evidence but the outcome may not show an incremental clinical benefit over available alternatives, or the lack of validation on surrogate outcomes prevents an assessment of potential clinical benefits.
- Cost only: in cases where cost-effectiveness is not demonstrated or cost is too high, as determined by the committee.
- Clinical and cost: in cases where a combination of both factors lead to the negative recommendation.

#### Reasons for negative recommendation

Each negative recommendation was classified according to the reason(s) stated in the recommendation document published by CADTH, referring to insufficient clinical data and/or unacceptable treatment costs.

#### Statistical analysis

Descriptive statistics were used to characterize continuous variables, using mean and standard deviation (SD) for normally distributed data and, for data that was not normally distributed, the median and range were calculated. Categorical variables are presented as percentages. We used the Mann-Whitney U test to compare medians for continues variables. Categorical variables were compared using the odds ratio (OR), conditional maximum likelihood estimates and Fisher’s Exact test. *P* values were two-sided and values < 0.05 were considered as statistically significant. If a DRD submission was labeled as a ‘resubmission’, we excluded the original submission from the statistical analysis, resulting in the analysis of only unique submissions, in order to preserve independence of statistical observation and to prevent skewing of the results by duplication of observations. Observations with missing data were excluded from the specific analysis. Correlation between prevalence and annual cost was tested through the use of Spearman Rank Correlation.

### Results

**Fig. 1:**
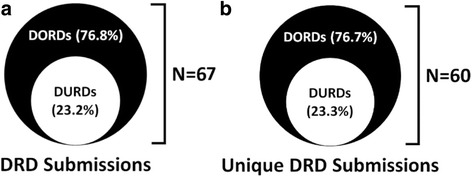
**a** Drug for rare diseases submissions, including resubmissions of drugs with same indication due to the availability of new evidence that may change the original recommendation. **b** Unique submissions of drugs for rare diseases, where we considered only the latest submission of drugs with multiple submissions for the same indication

In the study period of 2004 to 2016, we identified 67 submissions made to the CADTH CDR for drugs to treat diseases with a prevalence of ≤ 50 per 100,000 people, i.e. for DRDs, for which recommendations were issued. Of these, 16 (23.2%) were submissions for DURDs; the remaining 50 (76.8%) submissions were for DORDs. The relative proportions of the number of submissions made for DORDs and DURDs are illustrated in Fig. 1a. One submission in the DURD categories was a resubmission, and one a request for advice that included a reimbursement recommendation. Five submissions in the DORDs category were resubmissions, resulting in a total number of unique DRD submissions of 60, of which 14 (23.3%) are unique DURDs and 46 (76.7%) are unique DORDs.

**Fig. 2:**
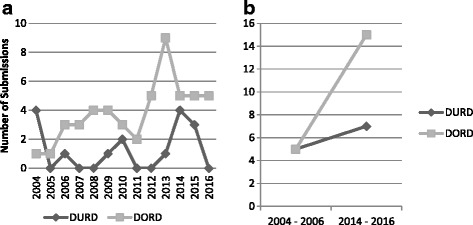
Time series of the annual number of DRD submission made to CDR over 12 years (for the period 2004 through 2016). **a** Number of annual submission for the entire period. **b** Illustration of the change in the number of submissions at the beginning 3 years of the period versus the last 3 years

The annual number of submissions for DRDs (including resubmissions) for the period 2004 through 2016 is presented in Fig. 2. From 2004 through 2011, the number of submissions per year varied between 1 and 4 for both DORDs and DURDs (Fig. 2). However, since 2011, the number of submissions for DORDs exhibited an increase (Fig. 2a). Figure 2b illustrates an almost 3-fold increase in the number of DORDs submitted since the inception of the CDR process, from 5 submissions in the first 3 years of the study period (from 2004 through to 2006) to 15 in the last 3 years (from 2014 through to 2016). By contrast, the number of submissions for DURDs has increased at a much lower rate, with five submissions from 2004 through to 2006 and seven submissions between 2014 through to 2016 (Fig. 2b). Therefore, the observed growth in the total number of DRD submissions during the study period appears to be primarily driven by an increase in DORDs submissions (Fig. 2b).

Comparison of the variables studied in DURD and DORD submissions is shown in Table 2, with all statistical analysis including only unique DRDs submission, and excluding original submissions of a resubmission. The proportion of therapeutic agents that were biologic molecules was significantly higher in DURDs compared with DORDs (78.6% versus 37%, respectively).

**Table 2:** Comparison of study variables in DURDs and DORDs submissions

Variable	DURD *N* = 14	DORD *N* = 46	Effect size (95% CI)	P value
Molecular structure				
Biologic, n (%)	11 (78.6)	17 (37.0)	OR = 6.06 (1.25 to 38.58)	**0.012**
Small molecule, n (%)	3 (21.4)	29 (63.0)		
Missing data, n (%)	0 (0)	0 (0)		
Number of clinical studies considered, median (range)	3 (3)	2 (13)	Median difference = 0 (0 to 1)	0.305
Missing data, n (%)	1 (7.1)	1 (2.2)		
Level of evidence considered				
At least one double blinded RCT, n (%)	8 (57.1)	42 (91.3)	OR = 0.13 (0.02 to 0.70)	**0.007**
At least one open label RCT, n (%)	0 (0)	3 (6.5)		
At least one non-randomized uncontrolled trial, n (%)	6 (42.9)	1 (2.2)		
Missing data, n (%)	0 (0)	0 (0)		
Size of largest study, median, n of patients (range)	59 (156)	167 (1134)	Median difference = −108 (−234 to −50)	**0.0001**
Missing data, n (%)	0 (0)	5 (10.9)		
Clinical study comparator				
No control, n (%)	5 (35.7)	1 (2.2)	OR = 23.11 (2.23 to 1207.19)	**0.0019**
Historical control, n (%)	1 (7.1)	0 (0)		
placebo, n (%)	7 (50.0)	36 (78.3)		
Active control, n (%)	1 (7.1)	9 (19.6)		
Missing data, n (%)	0 (0)	0 (0)		
Annual treatment cost per patient, median (range)	CAN$330,395 (CAN$934,000)	CAN$52,596 (CAN$429,858.5)	Median difference = CAN$243,787.75 (83,396 to 329,050)	***P*** **< 0.0001**
Missing data, n (%)	1 (7.1)	17 (37.0)		
Incremental cost per quality adjusted life-year, median (range)	CAN$2,680,000 (CAN$560,000)	CAN$ 165,923.5 (CAN$4,574,241)	NA	NA
Missing data, n (%)	12 (85.7)	24 (52.2)		
Recommendation				
Positive recommendation, n (%)	5 (35.7)	33 (71.7)	OR = 0.22 (0.05 to 0.91)	**0.025**
Negative recommendation, n (%)	9 (64.3)	13 (28.3)		
Missing data, n (%)	0 (0)	0 (0)		

*Bold *P* value indicates statistical significance

The total number of clinical studies considered in the CDR review for each submission was similar for DORDs and DURDs, ranging from 1 and 3 studies for the majority of submissions in both categories (85%). Nearly all submissions for DORDs (91.3%) contained data from at least one double-blind RCT, compared to only 57.1% of DURDs submissions, a statistically significant difference. Almost half of all DURDs submissions (42.9%) included data from non-randomized uncontrolled trials as their best level evidence, whereas only one submission (2.2%) in the DORD category contained a similarly low level of evidence. In addition to relying largely on non-randomized uncontrolled trial data, more than a third (35.7%) of DURDs submissions contained only evidence from clinical trials without a comparator, and one submission (7.1%) contained data generated with a historical control group. In contrast, only one submission (2.2%) in the DORDs category contained data from clinical studies without a comparator. We also found that half of DURDs submissions included evidence generated from clinical trials with placebo control. The median of the largest study size reported in each submission was statistically significantly smaller in the DURDs group (median = 59, range = 156) compared to the DORDs group (median = 167, range = 1134). The median difference in the size of the largest study in the DURD compared to DORDs was −108 (95%CI -234 to −50) and statistically significant. Cost information was available for 13 (92.9%) DURDs submissions and 29 (63.0%) DORDs submissions, with a median annual cost per patient of CAN$330,395 (range = CAN$934,000) for DURDs and CAN$52,596 (range = CAN$429,858) for DORDs respectively. The difference in annual cost per patient between DURDs and DORDs was statistically significant, with a median difference of CAN$243,787 (95%CI 83,396 to 329,050). Incremental cost per quality adjusted life-year was available for 2 (14.3%) DURDs submissions and 22 (47.8%) DORDs submissions, with a median inctemental cost per quality adjusted life-year of CAN$2,680,000 (range = CAN$560,000) for DURDs and CAN$165,923.5 (range = CAN$4,574,241.0). The median difference and 95% confidence interval for in the incremental cost per quality adjusted life-year between DURDs and DORDs was not calculated due to limited number of obsrvations in the DURDs. Overall, submissions for DURDs received more negative recommendations than DORD submissions (64.3% vs. 28.3%, respectively), and differences in the recommendation types were statistically significant.

**Fig. 3:**
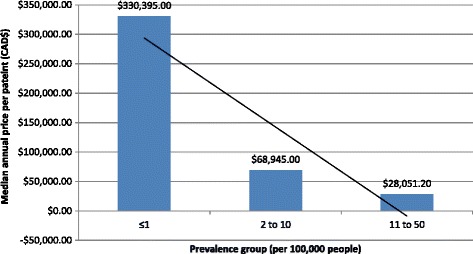
Average annual treatment cost per patient categorized by prevalence groups (CAN$)

The Spearman’ rank correlation coefficient (Rho) between disease prevalence and annual cost per patient was −0.51 (95%CI -0.71 to −0.25), with a statistically significant *P* value of 0.0003. Figure 3 presents a plot of all unique DRD submissions for which available annual treatment cost per patients were available, as per the methods section, (*N* = 42, missing = 18). The trend line for the annual cost increases sharply at the threshold of the disease prevalence range that corresponds to the definition we used for DURDs in this study.

Reasons for negative reimbursement recommendations are shown in Table 3. Insufficient clinical evidence was the most common reason for a negative recommendation for both DURDs and DORDs, followed by cost-related issues.

**Table 3:** Reasons for negative reimbursement recommendations for DURDs and DORDs

Reason	DURDs *N* = 9	DORDs *N* = 13
Clinical only, n (%)	8 (88.9)	8 (61.5)
Cost only, n (%)	1 (11.1)	1 (7.7)
Clinical and cost, n (%)	0 (0)	4 (30.8)

## Discussion

The current study, compared data from submissions for DRDs made to the CADTH CDR in Canada between 2004 through to 2016 to identify potential differentiating factors between DURDs and DORDs that could be utilized to optimize HTA of DRDs. A summary of the distinguishing characteristics between DORDs and DURDs are listed in Table 4 and discussed below.

**Table 4:** Summary of key similarities and differences between DURDs and DORDs identified in the present study

Similarities between DORDs and DURDs	
Number of studies	Submissions for DURDs and DORDs are similar in terms of the number of studies considered in the CDR review of clinical data
Overall reasons for negative recommendations	Insufficient clinical evidence was the most common reason for a negative recommendation for both DURDs and DORDs, followed by cost-related issues
Differences between DORDs and DURDs	
Growth in annual submission number	The steady growth in the total number of annual DRD submissions is predominantly attributable to growth in the number of annual submissions for DORDs, whereas the annual number of DURD submissions has risen only slightly
Molecular basis	DURDs are distinct in by being almost exclusively biologic molecules, whereas DORDs include a substantial proportion of small molecule-based therapies
Study size	Sample sizes for studies that support submissions for DURDs are generally smaller than those for DORDs
Study design	The majority of submissions for DURDs contained clinical data from non-randomized uncontrolled trials without comparator, whereas most DORD submissions included data from high-quality trial designs with active and/or placebo control arms and double-blinding
Cost	The average treatment costs of DURDS are generally substantially higher than those of DORDs
Recommendation type	Relatively more negative than positive reimbursement recommendations were issued for DURDs compared to DORDs
Reasons for negative recommendation	The rate of negative recommendations relating clinical reasons only was greater for DURDs than for DORDs

Our study has shown that there are significant differences between submissions for DURDs and DORDs. In particular, efficacy evidence submitted for DURDs tended to derive from smaller clinical studies, often of an uncontrolled design. Notwithstanding that more than half of DURD submissions did include a double-blind, randomized placebo-controlled trial, the quality of supporting clinical data was often cited as the reason for a higher rate of negative recommendations than for DORD submissions. These observations are likely a reflection of the difficulty in recruiting patients to clinical studies of ultra-rare diseases due to very low disease prevalence and suggest that the level of clinical evidence required for DURDs should be less exacting than that for DORDs. Our findings suggest that it may be inappropriate to apply the same appraisal standards to DURDs and DORDs. The current practice of using the same HTA appraisal processes for DURDs and DORDs could account for the relatively higher rate of negative reimbursement recommendations for DURD submissions observed in this study.

Thus, a distinct HTA process for DURDs that also considers benefits of a DURD which may not currently be captured in a cost-effectiveness analysis could be more appropriate to fully characterize the added value of a DURD. Several major international jurisdictions have recently introduced specific HTA frameworks for DRDs alongside established HTA processes for non-rare diseases [8–10]. In Canada, the CADTH CDR recommendation framework was changed in 2012 to accommodate DRD submissions under a special category that emphasizes unmet needs and severity of the condition [11]. We reported previously that, since this revised framework was adopted, no negative reimbursement recommendation due to lack of demonstrating cost-effectiveness or due to a high drug price alone were issued to a DRD [6]. Our current study provides further support for the feasibility of an HTA framework for DRDs that applies different appraisal criteria for DURDs and DORDs. At the same time, our findings question the need for a distinct HTA process to accommodate drugs that treat RDs with a prevalence of > 1 per 100,000 patients. However, whether the clinical evidence and cost-effectiveness of DORDs are more similar to that of drugs for non-rare diseases other than DURDs is yet to be determined. As is evident by the observation that 91.3% of the HTA submissions for DORDs contained data from double-blind RCTs, the barriers to generating sufficient clinical data in these small patient populations do not appear insurmountable. It could be argued that redefining a RD to align with the definition of an ultra-RD as a disease that affects ≤ 1 in 100,000 people would obviate the need for a distinct definition of ultra-RDs and their treatments as a sub-category of RDs, and would allow for the application of extant HTA frameworks developed to assess treatments for non-RDs for technologies targeted at diseases with a prevalence of > 1 per 100,000 individuals.

If DURDs were to be classed as a category of drugs that is distinct from DORDs, the definition of what constitutes a DURD would be a crucial factor. In this study, we defined DURDs as diseases that affect ≤ 1 in 100,000 people, which differs from the prevalence threshold cited in other published definitions (2 in 100,000 people) (8-10). However, we found that the disease prevalence in the DRD submissions examined for this study were generally classifiable around the 1 in 100,000. We, therefore, considered a prevalence threshold of 1 in 100,000 individuals the most appropriate and practicable cut-off for the definition of a DURD. The consideration of prevalence alone to define DURDs would nevertheless be problematic for several reasons, all of which apply to current definitions of DRDs. First, prevalence is essentially an arbitrary metric. Second, prevalence for a disease may vary among different geographic locations both within and among countries [12]. Third, reliable prevalence data for rare and ultra-rare diseases are difficult to generate due to high rates of underdiagnoses and diagnostic delay [13, 14]. Additional prescriptive characteristics are therefore often used to define RDs, such as a genetic etiology, an onset in childhood, and additional factors such as disease severity [5].

The parameters included in the definition of a DRD are of wider relevance: to date, DRDs have been considered by some stakeholder groups as being largely exempt from cost-related restrictions commonly placed on drugs for non-RDs, including ‘traditional’ cost-effectiveness thresholds [15–17]. Instead, the potential impact of treatment costs for DRDs are generally considered based on a total projected cost (i.e., a budget impact), an approach that has been criticized for failing to adequately account for the assessment of the relative value of such treatments [18, 19]. In our study, we found that higher disease prevalence was associated with lower treatment costs and that the average costs of DURDS were generally substantially higher than those of DORDs.

Rawson [7] reanalyzed the CADTH reimbursement recommendations we reported in our previous paper [6] to explore cost related differences of DRD with a prevalence of ≤ 1, between 1 and 10, and between 10 and 50 per 100,000 people and reported a slightly lower rate of negative recommendations compared to our findings in the current study However, his analysis was based on 55 CDR submissions made between 2004 and 2015. In comparison, the results of this current study included 60 CDR submissions in the period from 2004 through 2016. We included any CDR submission that received a reimbursement recommendation, including Requests for Advice that resulted in a published recommendation, and we excluded from our analysis the original DRD submission in case of the existence of a more recent resubmission; this was done to be able to elucidate true differences in DURD that are not biased by potential duplicate submissions. Consistent with Rawson’s findings [7], we also observed statistically significant differences in of the annual treatment cost and study size, and did not find a statistically significant difference in the number of trials included in the submission. The highly skewed distribution that describes the inverse relationship between prevalence and cost suggests that there is a dramatic increase in cost as diseases prevalence becomes very low. This finding is subject to some uncertainty, because cost information was unavailable in over one-third of the submissions that we examined; therefore, it is possible that a systematic avoidance of disclosing high DORDs prices may reduce the apparent discrepancy in the treatment costs of these drugs versus DURDs. In addition, we noted a higher proportion of biologic drugs in the DURDs submissions as compared to the DORDs. However, we were not able to elucidate the true cost difference in the development and manufacturing of biologics versus chemical drugs as opposed to factors related to demand, competition, and overall market opportunities. As such, we could not adjust for the potential impact of the higher proportion of biologics in DURDs versus DORDs. We did not compare the potential budget impact of DURDs to DORDs. However, the distinctly higher per-patient treatment cost of DURDs suggests that manufacturers are less likely to be guided by traditional cost-effectiveness thresholds when pricing treatments for ultra-rare diseases. This provides scope for the incorporation of a budget-impact based threshold into HTA decision frameworks specific for DURDs.

Although a distinct HTA review process for DURDs might lead to the expectation of improved access to DURDs, the same dilemma would face policy makers of not having access to high-level evidence to support the clinical effectiveness of a DURD. In their decision-making about the commissioning of a potentially high-cost life-long therapy, policy makers and public payers strive towards minimising uncertainty with respect to the clinical effectiveness and cost data. In addition, an ethical question might arise in that accepting low level evidence could expose patients to harm from potential adverse events whilst having little certainty of clinical benefits [20]. Also, it might be necessary to further explore potential opportunity cost that could be imposed on other disease areas as a result of increased positive reimbursement recommendation under a distinct DURDs HTA review process [21]. It might be necessary to explore new reimbursement models where a DURD is reimbursed with conditions of collecting and reporting back real-world data and reassessment of the clinical benefits would take place upon the generation of new evidence.

### Limitations

Our study has several limitations. First, we limited our review to submissions made to the CADTH CDR in Canada. Therefore, our results might not be generalizable to other jurisdictions, although it should be noted that the clinical evidence used to support reimbursement submissions in Canada is largely identical to that used in other countries, and Canadian pricing rules usually ensure that drug prices are within the range of international prices. Therefore, the CADTH CDR is a reasonable proxy for other HTA agencies, although similar studies of other HTA bodies would be needed to confirm our findings.

Second, the number of DRD submissions that we identified was relatively small and therefore decreases the robustness of the comparative statistics. However, this limitation reflects the nature of DRD reimbursement assessments: while the number of DRD reimbursement submissions has been continuously on the rise in recent years, there are still relatively few DRD submissions compared to submission for technologies that treat less rare diseases.

Third, as noted above, prevalence for a disease may vary across different geographic locations both within and across countries; therefore, a disease classified as rare in our study might not be considered rare elsewhere. Similarly, there might be diseases that are considered rare in some regions that were not classified as rare in our study.

Finally, as we used an explicit and arbitrary prevalence-based threshold to define DRDs in our study, application of a different threshold may have resulted in different findings. Therefore, our results might not be applicable to jurisdictions that have a substantively different definition of ‘rare disease’.

## Conclusions

DORDs and DURDs are similar in terms of the number of clinical studies used to support HTA submissions. In contrast, HTA submissions for DURDs differ from those for DORDs in some key areas: study sizes for DURDs are smaller, reliance on uncontrolled trials is more frequent, DURDs are more likely to be complex molecules (biologics), and the cost of DURDs is higher. These factors may all have contributed to a higher rate of negative reimbursement recommendations observed for DURDs compared to DORDs. Recognition of DURDs as a distinct subgroup of RDs may facilitate the development of HTA assessment processes that appropriately account for the inherent limitations that appear to be unique to DURDs. Based on the prevalence threshold applied in our study, we suggest that DURDs could be defined as diseases that affect ≤ 1 patients per 100,000 people, and that this prevalence threshold is combined with additional objective and descriptive criteria.

## Abbreviations

95%CI: 95% confidence interval
CADTH: Canadian agency for drugs and technologies in health
CAN$: Canadian dollar
CDEC: Canadian drug expert committee
CDR: Common drug review
DORD: Drug for other rare disease
DRD: Drugs for rare diseases
DURD: Drug for ultra-rare disease
HTA: Health technology assessment
OR: Odds ratio

## Footnotes

1 This applies to submissions made prior to 2012, after which a revised recommendation framework eliminated the ‘Do Not List at the Submitted Price’ category.
